# Chemical chaperon 4-phenylbutyrate protects against the endoplasmic reticulum stress-mediated renal fibrosis *in vivo* and *in vitro*

**DOI:** 10.18632/oncotarget.7904

**Published:** 2016-03-03

**Authors:** Shing-Hwa Liu, Ching-Chin Yang, Ding-Cheng Chan, Cheng-Tien Wu, Li-Ping Chen, Jenq-Wen Huang, Kuan-Yu Hung, Chih-Kang Chiang

**Affiliations:** ^1^ Graduate Institute of Toxicology, College of Medicine, National Taiwan University, Taipei, Taiwan; ^2^ Department of Pediatrics, National Taiwan University Hospital, Taipei, Taiwan; ^3^ Department of Medical Research, China Medical University Hospital, China Medical University, Taichung, Taiwan; ^4^ Department of Internal Medicine, College of Medicine, National Taiwan University, Taipei, Taiwan; ^5^ Department of Geriatrics and Gerontology, National Taiwan University Hospital, Taipei, Taiwan; ^6^ Superintendent's Office, National Taiwan University Hospital, Chu-Tung Branch, Taipei, Taiwan; ^7^ Department of Dentistry, Taipei Chang Gang Memorial Hospital, Chang Gang University, Taipei, Taiwan; ^8^ Department of Integrated Diagnostics & Therapeutics, National Taiwan University Hospital, Taipei, Taiwan

**Keywords:** sodium 4-phenylbutyrate, endoplasmic reticulum stress, unilateral ureteral obstruction, apoptosis, renal fibrosis

## Abstract

Renal tubulointerstitial fibrosis is the common and final pathologic change of kidney in end-stage renal disease. Interesting, endoplasmic reticulum (ER) stress is known to contribute to the pathophysiological mechanisms during the development of renal fibrosis. Here, we investigated the effects of chemical chaperon sodium 4-phenylbutyrate (4-PBA) on renal fibrosis *in vivo* and *in vitro*. In a rat unilateral ureteral obstruction (UUO) model, 4-PBA mimicked endogenous ER chaperon in the kidneys and significantly reduced glucose regulated protein 78 (GRP78), CCAAT/enhancer binding protein (C/EBP) homologous protein (CHOP), activating transcription factor 4 (ATF4), and phosphorylated JNK protein expressions as well as restored spliced X-box-binding protein 1 (XBP1) expressions in the kidneys of UUO rats. 4-PBA also attenuated the increases of α-smooth muscle actin (α-SMA), connective tissue growth factor (CTGF) protein expressions, tubulointerstitial fibrosis, and apoptosis in the kidneys of UUO rats. Moreover, transforming growth factor (TGF)-β markedly increased ER stress-associated molecules, profibrotic factors, and apoptotic markers in the renal tubular cells (NRK-52E), all of which could be significantly counteracted by 4-PBA treatment. 4-PBA also diminished TGF-β-increased CTGF promoter activity and CTGF mRNA expression in NRK-52E cells. Taken together, our results indicated that 4-PBA acts as an ER chaperone to ameliorate ER stress-induced renal tubular cell apoptosis and renal fibrosis.

## INTRODUCTION

Chronic kidney disease (CKD) progressed to end-stage renal disease is characterized by renal cell loss and diffuse fibrosis. Tubulointerstitial fibrosis is a common pathological presentation of CKD, characterized by tubular atrophy, mononuclear cell infiltration, and accumulation of myofibroblasts as well as dynamic remodeling of extracellular matrix (ECM) [1–3]. According to previous studies, transforming growth factor (TGF)-β has been regarded as a key regulator of renal fibrosis [4–6] and then incites connective tissue growth factor (CTGF) to induce the ECM induction and up-regulated the activities of profibrogenic factors in tubular cells, mesangial cells, and interstitial fibroblasts in fibrotic kidneys [7–10]. In light of these findings, the attenuation of CTGF has been considered as a potential target to prevent progressive renal fibrosis [11].

The endoplasmic reticulum (ER) stress is characterized by the accumulation of unfolded proteins in the ER lumen and is considered a key player in cellular stress responses, including the oxidative stress, metabolic stress, glucose starvation, elevated protein synthesis, and misfolding protein stress [12–15]. Glucose regulated protein 78 (GRP78) is one of the ER chaperone, is synthesized when cells are under stress, binds to the misfolding proteins to prevent them from aggregation, and assists these misfolded proteins to refold properly. Under overwhelming ER stress, three sensors including the activating transcription factor (ATF)-6, the inositol-requiring 1α (IRE1α) and the protein kinase RNA-like ER kinase (PERK) would release from GRP78 and play the protection roles against outcome stress [16]. ATF6 would be cleaved by Site-1 protease (S1P) and Site-2 protease (S2P) to release its cytoplasmic domain into the nucleus and induce expression of the ER-associated degradation and CCAAT/enhancer binding protein (C/EBP) homologous protein (CHOP) [17, 18]. Second, the released and activated IRE1α would be isolated and then splices the mRNA of X-box-binding protein 1 (XBP1), which encodes the ER chaperones to degrade or refold the misfolded proteins accumulated in the ER lumen. Third, homodimerized and phosphorylated PERK would phosphorylate eukaryotic initiation factor 2α (eIF2α) further to activate the activating transcription factor 4 (ATF4). ATF4 could transcriptionally activate many downstream targets, and is one of the transcription factor of collagen I [19]. These ER stress signal cascades may induce the molecular chaperones (such as heat shock protein, or GRP94), antioxidant enzymes and translational shutdown to prevent cells from apoptosis, inflammation and fibrosis [20–22].

4-phenylbutyrate (4-PBA), an aromatic fatty acid analog, is used to treat urea cycle disorders, because its metabolites offer an alternative pathway to the urea cycle to allow excretion of excess nitrogen [23]. Moreover, 4-PBA is also a histone deacetylase inhibitor [24, 25] and chemical chaperone [26], leading respectively to research into its use as an anti-cancer agent and in protein misfolding diseases such as cystic fibrosis [27]. Recently, 4-PBA also shows its therapeutic potential in many diseases models, including ischemia reperfusion injury, cystic fibrosis [27], and diabetes [28]. In our previous report [29], we successfully demonstrated the activation of ER stress, which actively contributed to the development of renal fibrosis in the rat unilateral ureteral obstruction (UUO) kidney. Therefore, we hypothesized that administration of 4-PBA, through its chemical chaperone property, may alleviate renal fibrosis. In this study, we designed the experiments to investigate the protective effect of 4-PBA on renal fibrosis both *in vivo* and *in vitro.*

## RESULTS

### 4-PBA reduced overwhelming ER stress and profibrogenic signals in rat UUO kidneys

**Figure 1:**
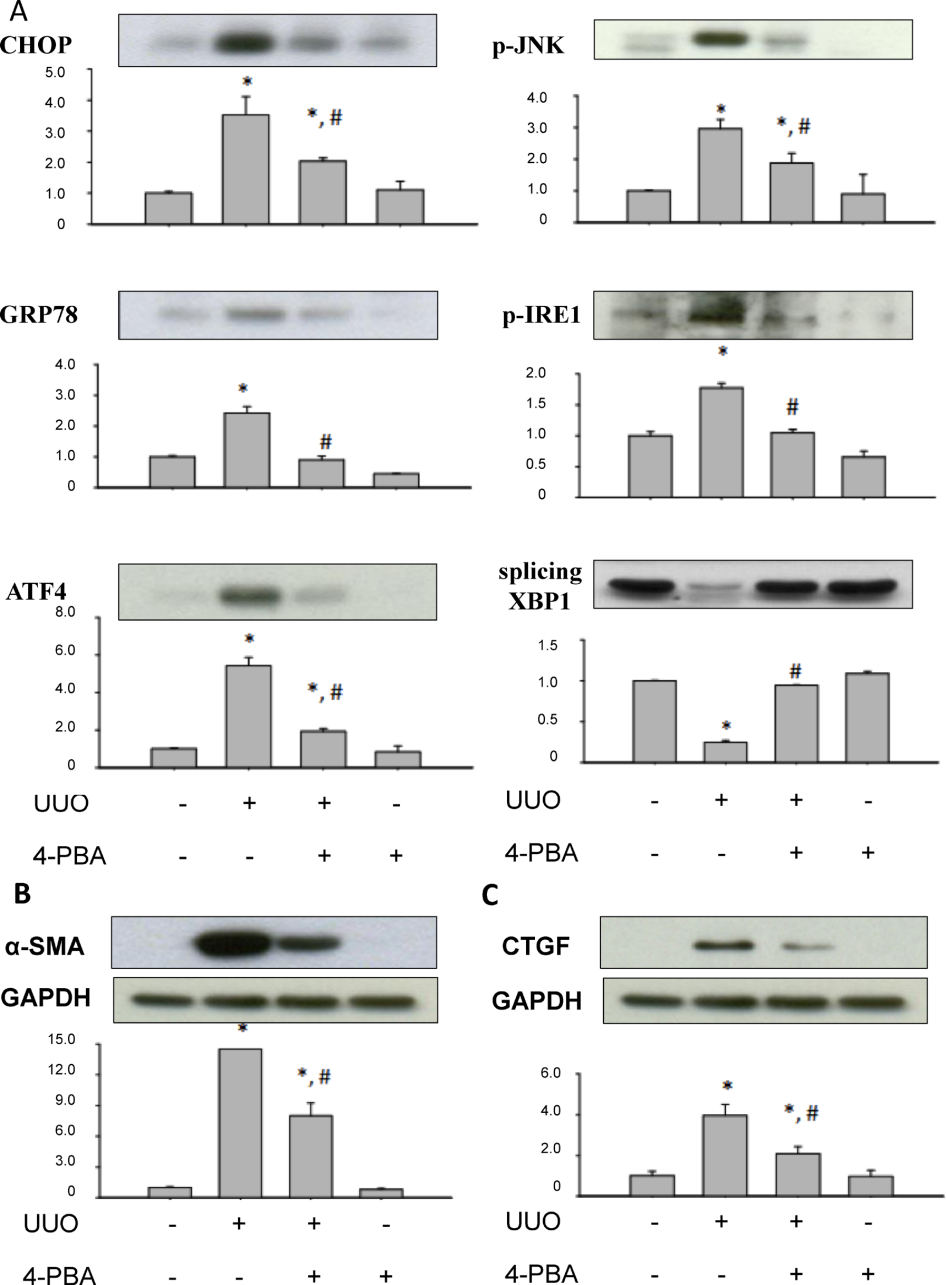
4-PBA attenuated overwhelming ER stress response and profibrotic markers in kidneys of UUO rats 4-PBA treatment (500 mg/kg, twice a day) and UUO rats were dissection after 14 days. **A.** UUO induced overwhelming ER stress-related signals, including CHOP, GRP78, ATF4, JNK phosphorylation, IRE-1 phosphorylation, and splicing of XBP1 protein, which could be reversed by 4-PBA treatment. **B** and **C.** The profibrotic marker and transcriptional factor, α-SMA and CTGF, were induced in UUO kidneys, which could be attenuated by 4-PBA. Protein expression levels were quantified by densitometry. X-axis means the groups of treatment, and the Y-axis represents the fold of induction as compared with sham group. Data are presented as mean ± SEM (n = 4). *P< 0.05 versus sham group, #*P* < 0.05 versus UUO group.

4-PBA, a chemical chaperon, ameliorated UUO-caused ER stress-associated GRP78, CHOP, ATF4, and phosphorylated JNK protein expressions. 4-PBA also restored UUO-suppressed splicing XBP-1 protein level in the pathological kidney (Figure 1A). Furthermore, α-smooth muscle actin (α-SMA), the representative epithelial-mesenchymal transition (EMT) marker and CTGF, the profibrotic factor, both protein expressions were induced in UUO rats and significantly attenuated by 4-PBA administration (Figure 1B and 1C).

### 4-PBA attenuated interstitial damage, collagen deposition, and cell apoptosis in UUO kidneys

**Figure 2:**
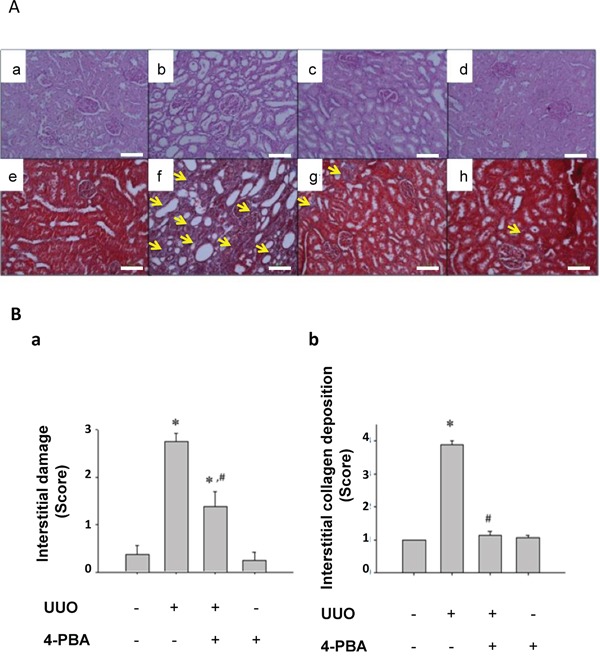
4-PBA ameliorated interstitial damage and collagen deposition in UUO rat kidneys Interstitial damage was detected by PAS staining. Sham-operative rat kidney showed normal tubular conformation without interstitial damage **A.** (a). UUO rat kidney revealed severe interstitial damage (b). 4-PBA treatment (500 mg/kg, twice a day) attenuated tubular interstitial damage induced by UUO (c). 4-PBA treatment did not affect the tubular conformation in sham-operative rat kidney (d). Moreover, the collagen deposition was detected by Masson trichrome staining. UUO rat kidney (f) showed marked collagen deposition as compared with sham-operative kidney (e). 4-PBA treatment attenuated the collagen deposition induced by UUO (g). 4-PBA treatment did not affect the collagen deposition in sham-operative kidney (h). The pathological scores of interstitial damage and collagen deposition **B.** (a and b, respectively) were quantified and presented as mean ± SEM values (n = 3). *P< 0.05 versus sham group, #*P* < 0.05 versus UUO group. The yellow arrow indicated the blue stain of Masson trichrome. The white scale bar represents 100 μm.

**Figure 3:**
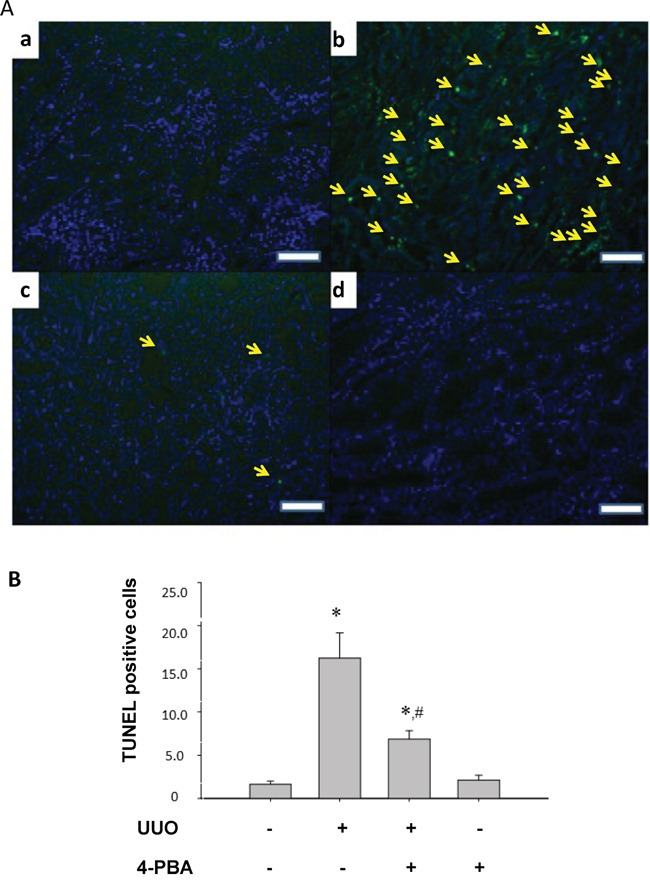
4-PBA attenuated apoptotic renal cell in kidneys of UUO rats The apoptotic cells in UUO rats were evaluated by TUNEL assay. Sham-operative kidney showed no positive nuclear staining for TUNEL **A.** (a). UUO kidney showed a significant increase in TUNEL-positive staining at day 14 (b). 4-PBA treatment (500 mg/kg, twice a day) attenuated UUO-induced renal cell apoptosis (c). 4-PBA-treated sham-operative kidney showed no positive TUNEL staining (d). The TUNEL-positive staining cells in the kidneys were calculated as described in Materials and Methods **B.** Data are presented as mean ± SEM values (n = 3). *P< 0.05 versus the sham control group. #*P*< 0.05 versus the UUO group. The white scale bar represents 100 μm.

UUO induced tubulointerstitial damage was examined by periodic acid-Schiff (PAS) and Masson's trichrome staining. Severe tubule atrophy and widened interstitial space were observed in UUO-treated kidney by PAS staining (Figure 2A–b). Numerous collagen deposition in the UUO-treated kidney was showed by Masson's trichrome staining (Figure 2A–f). 4-PBA treatment significantly reversed UUO-caused tubule atrophy, widened interstitial (Figure 2A–c), and collagen deposition (Figure 2A–g). The score of interstitial damage and collagen deposition were quantified (Figure 2B). 4-PBA also ameliorated renal tubular cell apoptosis which occurred in UUO-treated rats (Figure 3).

### 4-PBA attenuated overwhelming ER stress, renal tubular cell apoptosis, and profibrogenic factors induced by TGF-β in NRK-52E cells

**Figure 4:**
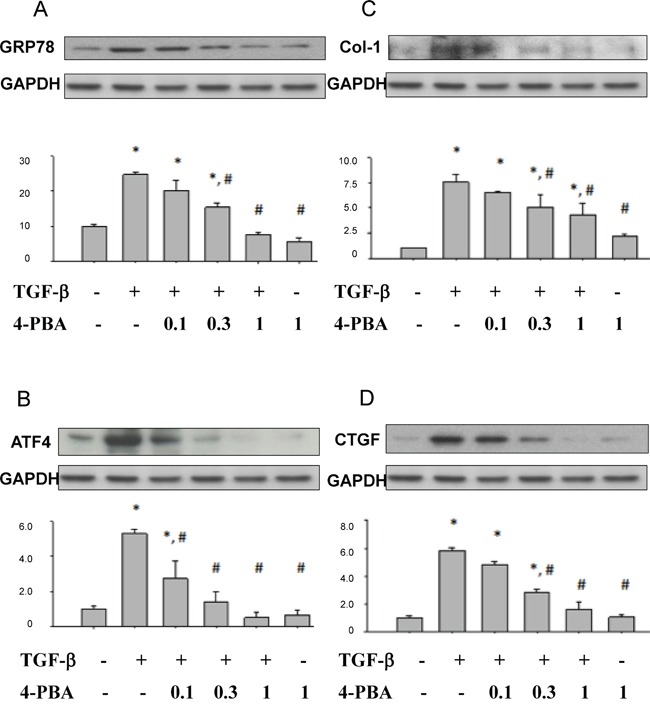
4-PBA attenuated ER stress-related molecules and fibrosis markers induced by TGF-β in renal tubular NRK52E cells NRK 52E cells were treated with 5 ng/ml TGF-β and 0.1 to 1 mM 4-PBA for 24 hours. Protein expressions of **A.** GRP78, **B.** ATF4, **C.** collagen-I, and **D.** CTGF were detected by Western blotting. Protein expression levels were quantified by densitometry. X-axis means the groups of treatment, and the Y-axis represents the fold of induction as compared with sham group. Data are presented as mean ± SEM values (n = 3) for triplication. *P< 0.05 versus control group. #*P* < 0.05 versus TGF-β group.

**Figure 5:**
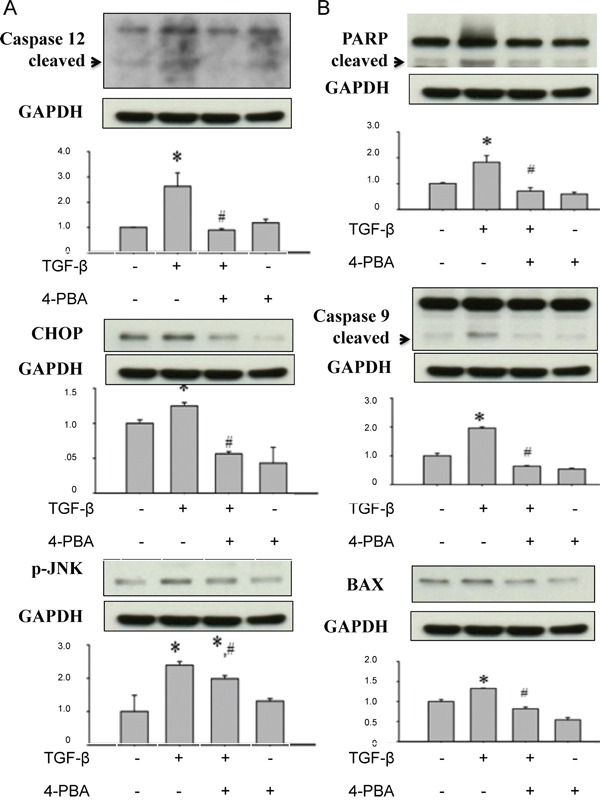
4-PBA attenuated ER stress-related apoptosis-associated molecules induced by TGF-β in renal tubular NRK52E cells NRK 52E cells were treated with 5 ng/ml TGF-β and 1 mM 4-PBA for 24 hours. **A.** TGF-β also increased caspase12 cleavage, CHOP protein expression, and JNK phosphorylation in NRK-52E cells, which signals were correlated with ER stress-related cell apoptosis, were significantly attenuated by 4-PBA. **B.** 4-PBA significantly attenuated the TGF-β-increased protein expressions of mitochondria-related apoptosis signals, including caspase 9, Bax, and PARP in NRK-52E cells. Protein expression levels were quantified by densitometry analysis. X-axis means the groups of treatment, and the Y-axis represents the fold of induction as compared with sham group. Data are presented as mean ± SEM values (n = 3) for triplication. *P< 0.05 versus control group. #*P* < 0.05 versus TGF-β group.

**Figure 6:**
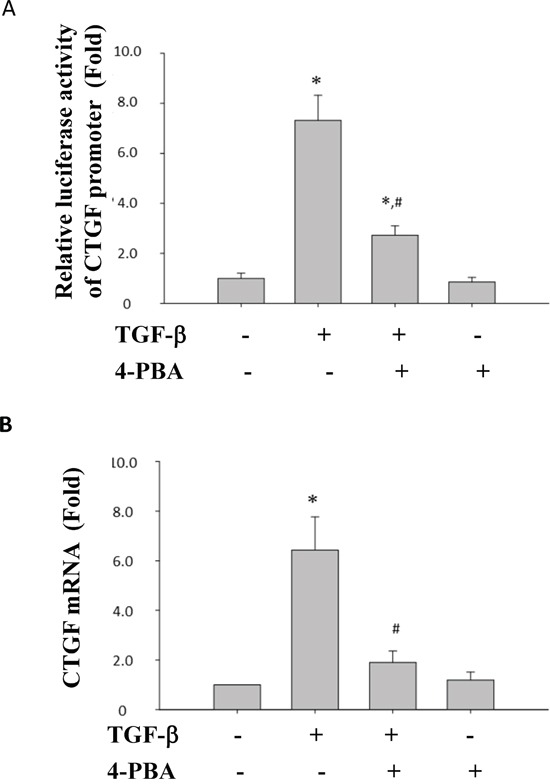
4-PBA inhibited TGF-β-induced CTGF promoter activity and CTGF mRNA expression in NRK52E cells The wild-type CTGF promoter (−747/+214) luciferase construct (pGL3-CTGF-Luc) was transfected into NRK-52E cells. 4-PBA was added 1 h prior to treatment of TGF-β. The relative luciferase activity were showed in **A.** NRK 52E cells were treated with 5 ng/ml TGF-β and 1 mM 4-PBA for 24 hours. mRNA expression of CTGF was analyzed by real-time PCR. **B.** Data are presented as mean ± SEM values (n = 3) for triplication. *P< 0.05 versus control group. #*P* < 0.05 versus TGF-β group.

Lovisa and colleagues recently reported that preventing the acquisition of an epithelial-mesenchymal transition (EMT) program in injured renal tubular epithelial cells (TECs) results in the preservation of functional TECs and organ function [30]. Inspired by their works, we investigated the effects of 4-PBA on TGF-β-induced injury and renal fibrosis in rat kidney epithelial NRK52E cells. As shown in Figure 4, 4-PBA attenuated TGF-β-induced ER stress-associated GRP78, ATF-4 and profibrogenic-related CTGF, collagen-I protein expression in a dose dependent manner. 4-PBA also diminished TGF-β-caused cell apoptosis signaling pathway including caspase12 cleavage, CHOP protein expression, and JNK phosphorylation in NRK-52E cells (Figure 5A). Moreover, 4-PBA significantly attenuated the TGF-β-increased caspase9, Bax, and PARP which represent intrinsic apoptotic pathway in NRK-52E cells (Figure 5B). In addition, 4-PBA significantly reduced the TGF-β-induced CTGF promoter activity (Figure 6A) and CTGF mRNA expression (Figure 6B) in NRK-52E cells indicating that 4-PBA possibly reversed fibrosis through CTGF transcriptional regulation. These results suggest that 4-PBA ameliorated TGF-β-caused overwhelming ER stress response and profibrogenic factor expression in renal cells.

## DISCUSSION

In this study, we demonstrated for the first time that 4-PBA is capable of attenuating the UUO- and TGF-β- induced renal tubular cell fibrosis and apoptosis *in vitro* and *in vivo*. 4-PBA, a chemical ER chaperone, ameliorated the ER stress, interstitial damage, collagen deposition, and cell apoptosis in UUO rat kidneys. 4-PBA also inhibited the TGF-β-induced ER stress and profibrogenic factors expression in renal tubular epithelial cells. These results suggest that 4-PBA may possess the potential therapeutic effect for progressive renal failure by ameliorating tubular fibrosis and apoptosis.

Our previous report has revealed that renal tubulointerstitial fibrosis and tubular cell apoptosis in UUO rat kidneys might be associated with ER stress, which includes elevated expression of GRP78, ATF6, PERK, and IRE-1 [29]. Tubulointerstitial fibrosis and cell apoptosis were considered to be associated with end-stage renal failure and progressive renal loss [31, 32]. Furthermore, Kawakami and colleagues recently indicated that indoxyl sulfate, a typical uremic toxin, would suppress the proliferation of human proximal tubular cells via the ER stress-dependent signaling pathway [33]. Uremic toxin accumulation may contribute to the deterioration of renal function in progressive chronic kidney disease. These findings suggested that ER stress-related signaling pathways are possible targets for retarding chronic kidney disease progression. In the present study, 4-PBA significantly attenuated UUO-induced renal GRP78, CHOP, ATF4, and phosphorylated JNK protein expressions and reversed UUO-reduced renal splicing XBP1 protein expression. 4-PBA also lowered the expressions of CTGF, α-SMA and improve the phenomenon of fibrosis in UUO rat kidneys. These results suggest that 4-PBA acts as an ER chaperone to ameliorate the ER stress and renal fibrosis.

The signals of TGF-β and its downstream cascade play key roles in activating cellular pathological mechanisms for renal tubulointerstitial fibrosis, including expression of pro-fibrotic genes and activation of interstitial cells [6, 34]. Members of the TGF-β family which bind to type I and type II serine/threonine kinase receptors would initiate intracellular signals through activation of Smad proteins [35, 36]. Activation of Smad protein family would proceed TGF-β signaling and regulate the promoter activities of TGF-β targeted genes [35, 36]. TGF-β could induce apoptosis in many cell types, including renal cells [37, 38], resulting in tubular degeneration and tubular atrophy. TGF-β receptor activation leads to activation of Smad3 which is required for the up-regulation of pro-apoptotic signaling mediators, death-associated protein kinase (DAPK) and Smad7 [39]. TGF-β activates pro-apoptotic MAPK p38 and JNK cascades which trigger Bad protein induction, caspases activation, and apoptosis in podocytes [40]. TGF-β-induced EMT also contributes to renal tubular atrophy, renal interstitial myofibroblast generation, and concomitant tubulointerstitial fibrosis [40]. The elevated expressions of TGF-β1 and pro-fibrotic mediators have been demostrated in UUO rat kidneys [41]. TGF-β also possessed the ability to convert tubular epithelial cells and fibroblasts into activated myofibroblasts, which may be responsible for the increased deposition of interstitial matrix [41]. Previous studies have revealed that CTGF plays a critical role in TGF-β-dependent tubulointerstitial fibrosis [42, 43]. Induction of CTGF by TGF-β would trigger the up-regulation of collagen type I in mesangial cells and fibroblasts. In recognition of previous study, we elucidate the roles of 4-PBA on overwhelming ER stress and profibrogenic factors (especially TGF-β and CTGF) signals. We found that 4-PBA not only suppressed the overwhelming ER stress, but also ameliorated the fibrotic pathology in UUO model and in TGF-β-induced renal tubular cells. 4-PBA also attenuated the TGF-β-increased CTGF promoter activity and CTGF mRNA expression in renal tubular cells.

In conclusion, we demonstrated for the first time that chemical chaperon 4-PBA effectively protects against the ER stress-mediated renal fibrosis both in UUO rat model and in TGF-β-treated fibrotic model. These findings suggest that 4-PBA may be a potential therapeutic option in the prevention or treatment of renal fibrosis.

## MATERIALS AND METHODS

### Experimental animals

All animal care and experimental procedures were approved by the Institutional Animal Care and Use Committee, and the care or use of laboratory animals were conducted in accordance with the guidelines of the Animal Research Committee of the College of Medicine, National Taiwan University. Male Wistar rats (Lasco, Taipei, Taiwan) weighing 150–200 g were housed in temperature-controlled conditions under a light/dark photo cycle with food and water supplied *ad libitum*. Following anesthesia with pentobarbital sodium (50 mg/kg, i.p.), the left ureter was ligated at the ureteropelvic junction with 4-0 silk through a left flank incision (UUO model). A control group of rats were subjected to sham operations that were identical to those for the rats with UUO, except that the ureters were not ligated.

### Experimental protocols for UUO

Four groups of rats (*n* = 20) were used. UUO rats were treated with either PBS (UUO group, *n* = 5) or 4-PBA (UUO/4-PBA group, *n* = 5) was administered two times a day in two divided doses (500 mg/kg for 8am and 8pm, total 1 g/kg/day, Sigma, St Louis, MO, USA) by oral gavage after the animal had recovered from surgery and general anesthesia through day 0 to day 13. The selection of 4-PBA dosages was based on the previous reports [28, 44] and our preliminary experiments [29]. Sham-operative rats consisted of five age-matched rats in each group. At day 14, UUO and sham control kidneys were divided into two parts. The first part was fixed in 10% neutral-buffered formalin for pathological examination and the second part was quickly frozen in liquid nitrogen and stored at −70°C for protein and RNA extraction.

### Semiquantitative assessment of renal fibrosis

Tubulointerstitial damage was graded in PAS-stained sections on a scale from 0 to 4 (0, no changes; 1, changes affecting <25%; 2, changes affecting 25 to 50%; 3, changes affecting 50 to 75%; 4, changes affecting 75 to 100% of the section). For further analysis of the degree of interstitial collagen deposition, Masson's trichrome-stained sections were graded (0, no staining; 1, <25% staining; 2, 25 to 50% staining; 3, 50 to 75% staining; 4, 75 to 100% staining of the section). Twenty cortical tubulointerstitial fields that were randomly selected at 400X magnification were assessed in each rat, and the average for each group then was analyzed.

### Cell culture and chemical treatment

Normal rat renal proximal tubular epithelial cells (NRK-52E) were purchased from American Type Culture Collection (ATCC) and cultured with Dulbecco's modified Eagle's medium and 5% fetal calf serum. Cells were treated with 4-PBA (0.1~1 mM) and TGF-β (5 ng/ml; R&D Systems, Minneapolis, MN, USA) in 0.1 or 0.5% FBS-added medium at the indicated dosages and time points. The selection of 4-PBA concentration was based on the previous study and our preliminary experiments [45].

### Immunoblotting analysis

NRK-52E cells were lysed in RadioImmunoPrecipitation Assay (RIPA) buffer. Whole-cell lysates were subsequently centrifuged at 13,000 ×*g* for 30 min, and cytosolic proteins were collected. Electrophoresis, immunoblotting, and detection were performed as described previously [46]. The relative values of each protein were normalized with GAPDH from the samples. The following primary antibodies were used in this study: CCAAT/enhancer binding protein homologous protein (CHOP), (Santa Cruz Biotechnology, Santa Cruz, CA, USA); phosphor eIF2α, poly (ADP-ribose) polymerase (PARP), caspase 9 (Cell Signaling Technology, Danvers, MA, USA); caspase 12, activating transcription factor (ATF)-4, phospho-Jun N-terminal kinase (JNK) (Abcam, Cambridge, MA, USA); and Bax (Millipore Technology, Billerica, MA, USA).

### Real-time RT-PCR

Real-time reverse-transcription PCR was performed in the Bio-Rad iQ5 detection system. Total RNA (5 μg) was reverse transcribed with the Promega reverse transcriptase reagent mix. The RT reaction products were diluted to the volumes of 10 μl for GAPDH, and 1 μl aliquots were used as template for amplification using the iQ SYBR Green supermix reagent (Bio-Rad, Hercules, CA, USA) and gene-specific primers. The primer sets for connective tissue growth factor (CTGF; forward, CAGGCTGGAGAAGCAGAGTCGT; reverse, CTGGTGCAGCCAGAAAGCTCAA), and GAPDH (forward, TGGCACAGTCAAGGCTGAGA; reverse, CTTCTGAGTGGCAGTGATGG) were used, and CTGF expression was normalized by GAPDH signals with the ΔC_T_ method.

### Construction of CTGF promoter-luciferase reporter plasmids

The CTGF promoter (−747/+214) luciferase construct (pGL3-CTGF-Luc) was provided by Dr M.-L. Kuo (National Taiwan University) [47]. Briefly, a 962-base pair (bp) fragment representing the 5′ upstream region of the CTGF gene (−747 to 214 bp), based on GenBank accession number AL354866, was generated by PCR. The initiation site of transcription was labeled as +1. This fragment was ligated into the firefly luciferase (Rluc) reporter vector, pGL3-basic (Promega, Madison, WI, USA), and was designated pGL3-CTGF, then verified by sequencing. Luciferase activities were quantified with the Dual-Luciferase Reporter Assay System (Promega, Madison, WI, USA).
